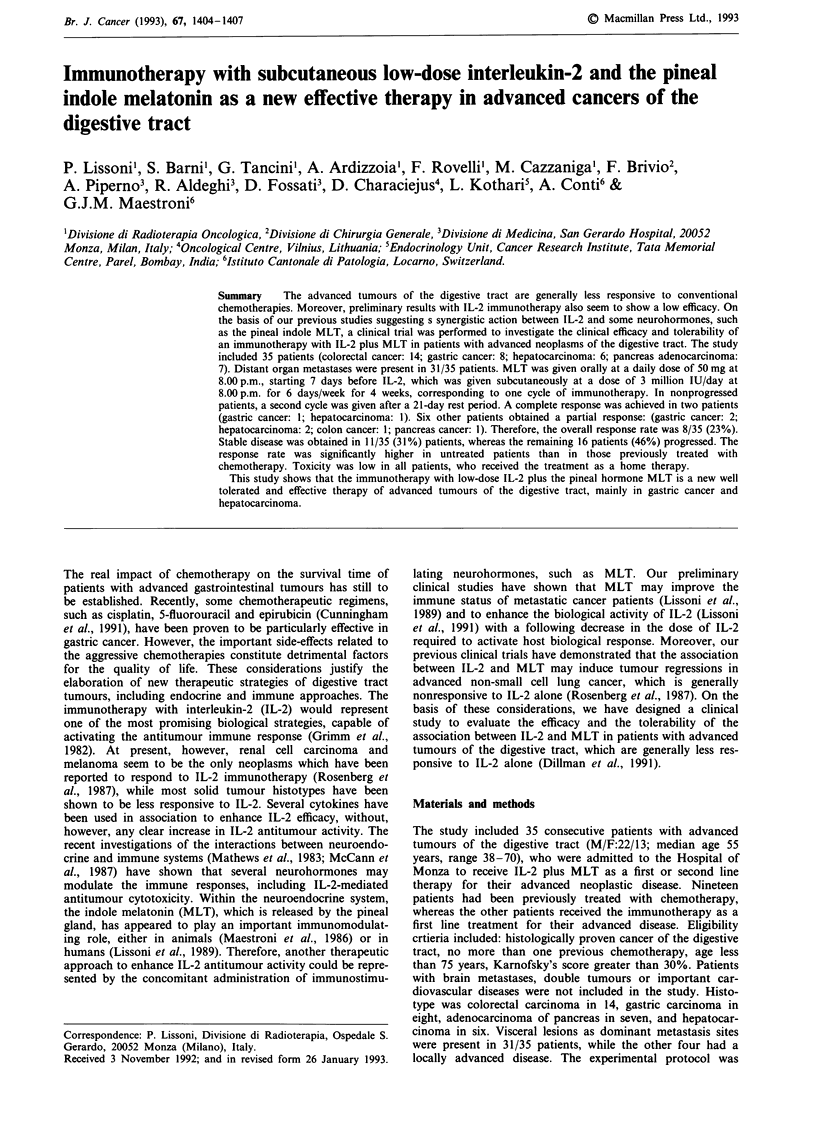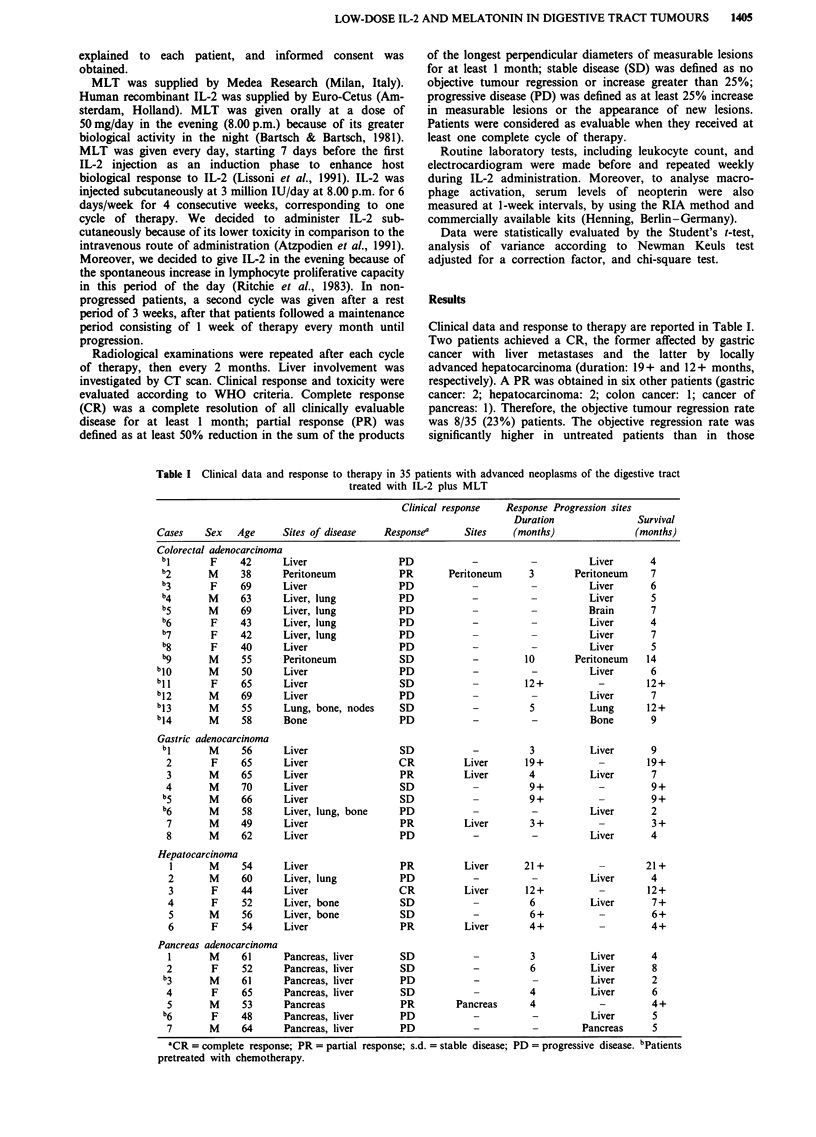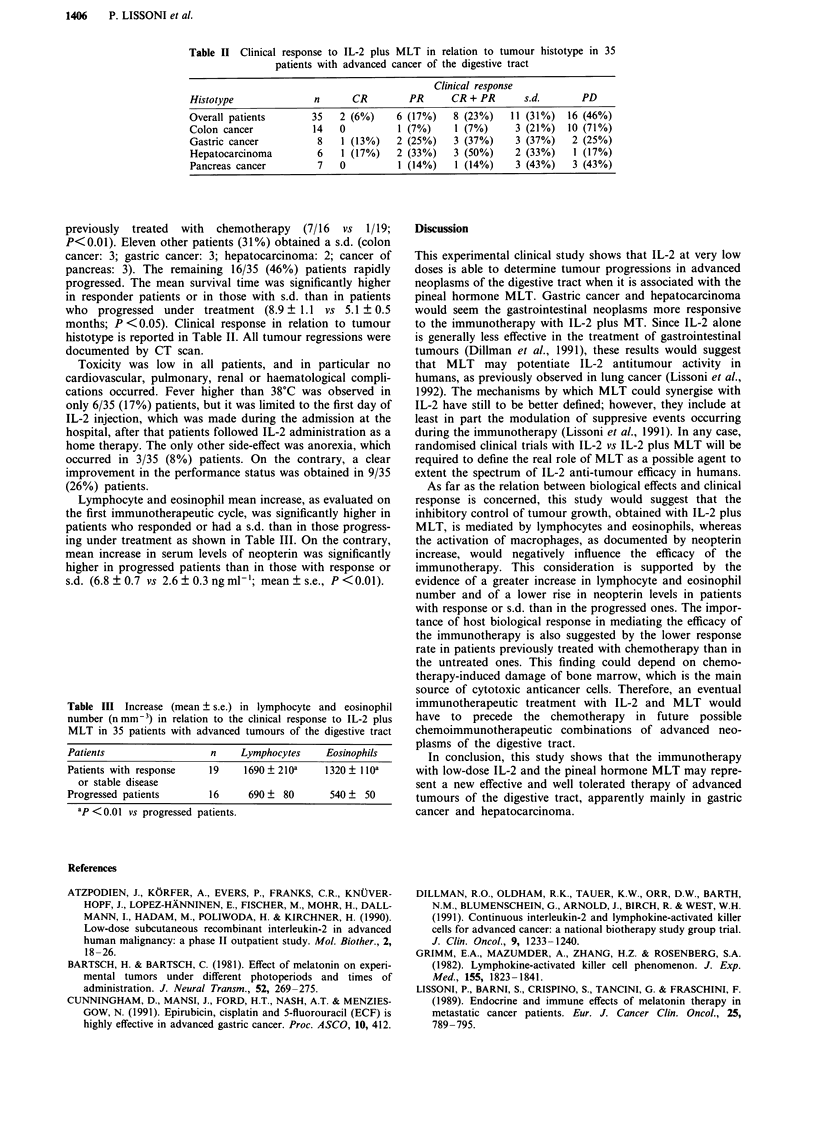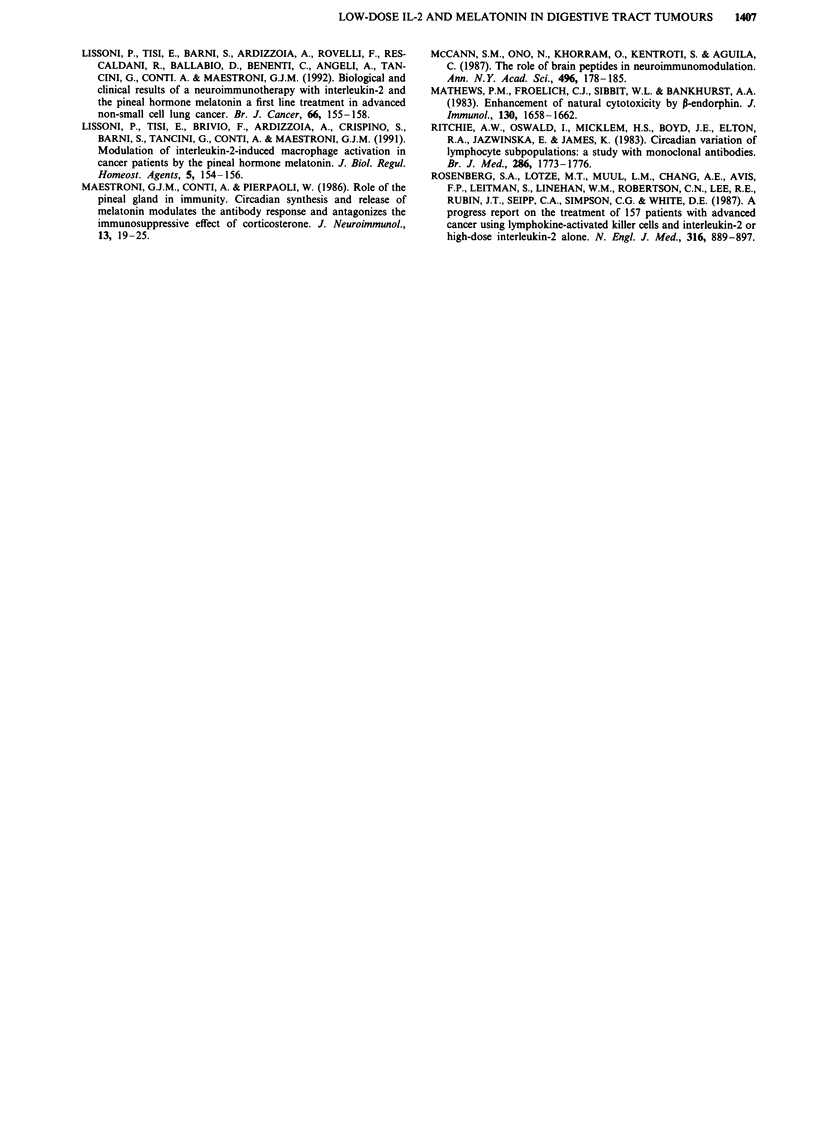# Immunotherapy with subcutaneous low-dose interleukin-2 and the pineal indole melatonin as a new effective therapy in advanced cancers of the digestive tract.

**DOI:** 10.1038/bjc.1993.260

**Published:** 1993-06

**Authors:** P. Lissoni, S. Barni, G. Tancini, A. Ardizzoia, F. Rovelli, M. Cazzaniga, F. Brivio, A. Piperno, R. Aldeghi, D. Fossati

**Affiliations:** Divisione di Radioterapia Oncologica, San Gerardo Hospital, Milan, Italy.

## Abstract

The advanced tumours of the digestive tract are generally less responsive to conventional chemotherapies. Moreover, preliminary results with IL-2 immunotherapy also seem to show a low efficacy. On the basis of our previous studies suggesting s synergistic action between IL-2 and some neurohormones, such as the pineal indole MLT, a clinical trial was performed to investigate the clinical efficacy and tolerability of an immunotherapy with IL-2 plus MLT in patients with advanced neoplasms of the digestive tract. The study included 35 patients (colorectal cancer: 14; gastric cancer: 8; hepatocarcinoma: 6; pancreas adenocarcinoma: 7). Distant organ metastases were present in 31/35 patients. MLT was given orally at a daily dose of 50 mg at 8.00 p.m., starting 7 days before IL-2, which was given subcutaneously at a dose of 3 million IU/day at 8.00 p.m. for 6 days/week for 4 weeks, corresponding to one cycle of immunotherapy. In nonprogressed patients, a second cycle was given after a 21-day rest period. A complete response was achieved in two patients (gastric cancer: 1; hepatocarcinoma: 1). Six other patients obtained a partial response: (gastric cancer: 2; hepatocarcinoma: 2; colon cancer: 1; pancreas cancer: 1). Therefore, the overall response rate was 8/35 (23%). Stable disease was obtained in 11/35 (31%) patients, whereas the remaining 16 patients (46%) progressed. The response rate was significantly higher in untreated patients than in those previously treated with chemotherapy. Toxicity was low in all patients, who received the treatment as a home therapy. This study shows that the immunotherapy with low-dose IL-2 plus the pineal hormone MLT is a new well tolerated and effective therapy of advanced tumours of the digestive tract, mainly in gastric cancer and hepatocarcinoma.


Br. J. Cancer (1993), 67, 1404–1407
© Macmillan Press Ltd., 1993

### Immunotherapy with subcutaneous low-dose interleukin-2 and the pineal indole melatonin as a new effective therapy in advanced cancers of the digestive tract

P. Lissoni[1], S. Barni[1], G. Tancini[1], A. Ardizzoia[1], F. Rovelli[1], M. Cazzaniga[1], F. Brivio[2], A. Piperno[3], R. Aldeghi[3], D. Fossati[3], D. Characiejus[4], L. Kothari[5], A. Conti[6] & G.J.M. Maestroni[6]

[1]*Divisione di Radioterapia Oncologica,* [2]*Divisione di Chirurgia Generale,* [3]*Divisione di Medicina, San Gerardo Hospital, 20052 Monza, Milan, Italy;* [4]*Oncological Centre, Vilnius, Lithuania;* [5]*Endocrinology Unit, Cancer Research Institute, Tata Memorial Centre, Parel, Bombay, India;* [6]*Istituto Cantonale di Patologia, Locarno, Switzerland.*

**Summary** The advanced tumours of the digestive tract are generally less responsive to conventional chemotherapies. Moreover, preliminary results with IL-2 immunotherapy also seem to show a low efficacy. On the basis of our previous studies suggesting s synergistic action between IL-2 and some neurohormones, such as the pineal indole MLT, a clinical trial was performed to investigate the clinical efficacy and tolerability of an immunotherapy with IL-2 plus MLT in patients with advanced neoplasms of the digestive tract. The study included 35 patients (colorectal cancer: 14; gastric cancer: 8; hepatocarcinoma: 6; pancreas adenocarcinoma: 7). Distant organ metastases were present in 31/35 patients. MLT was given orally at a daily dose of 50 mg at 8.00 p.m., starting 7 days before IL-2, which was given subcutaneously at a dose of 3 million IU/day at 8.00 p.m. for 6 days/week for 4 weeks, corresponding to one cycle of immunotherapy. In nonprogressed patients, a second cycle was given after a 21-day rest period. A complete response was achieved in two patients (gastric cancer: 1; hepatocarcinoma: 1). Six other patients obtained a partial response: (gastric cancer: 2; hepatocarcinoma: 2; colon cancer: 1; pancreas cancer: 1). Therefore, the overall response rate was 8/35 (23%). Stable disease was obtained in 11/35 (31%) patients, whereas the remaining 16 patients (46%) progressed. The response rate was significantly higher in untreated patients than in those previously treated with chemotherapy. Toxicity was low in all patients, who received the treatment as a home therapy.

This study shows that the immunotherapy with low-dose IL-2 plus the pineal hormone MLT is a new well tolerated and effective therapy of advanced tumours of the digestive tract, mainly in gastric cancer and hepatocarcinoma.

The real impact of chemotherapy on the survival time of patients with advanced gastrointestinal tumours has still to be established. Recently, some chemotherapeutic regimens, such as cisplatin, 5-fluorouracil and epirubicin (Cunningham *et al.*, 1991), have been proven to be particularly effective in gastric cancer. However, the important side-effects related to the aggressive chemotherapies constitute detrimental factors for the quality of life. These considerations justify the elaboration of new therapeutic strategies of digestive tract tumours, including endocrine and immune approaches. The immunotherapy with interleukin-2 (IL-2) would represent one of the most promising biological strategies, capable of activating the antitumour immune response (Grimm *et al.*, 1982). At present, however, renal cell carcinoma and melanoma seem to be the only neoplasms which have been reported to respond to IL-2 immunotherapy (Rosenberg *et al.*, 1987), while most solid tumour histotypes have been shown to be less responsive to IL-2. Several cytokines have been used in association to enhance IL-2 efficacy, without, however, any clear increase in IL-2 antitumour activity. The recent investigations of the interactions between neuroendocrine and immune systems (Mathews *et al.*, 1983; McCann *et al.*, 1987) have shown that several neurohormones may modulate the immune responses, including IL-2-mediated antitumour cytotoxicity. Within the neuroendocrine system, the indole melatonin (MLT), which is released by the pineal gland, has appeared to play an important immunomodulating role, either in animals (Maestroni *et al.*, 1986) or in humans (Lissoni *et al.*, 1989). Therefore, another therapeutic approach to enhance IL-2 antitumour activity could be represented by the concomitant administration of immunostimulating neurohormones, such as MLT. Our preliminary clinical studies have shown that MLT may improve the immune status of metastatic cancer patients (Lissoni *et al.*, 1989) and to enhance the biological activity of IL-2 (Lissoni *et al.*, 1991) with a following decrease in the dose of IL-2 required to activate host biological response. Moreover, our previous clinical trials have demonstrated that the association between IL-2 and MLT may induce tumour regressions in advanced non-small cell lung cancer, which is generally nonresponsive to IL-2 alone (Rosenberg *et al.*, 1987). On the basis of these considerations, we have designed a clinical study to evaluate the efficacy and the tolerability of the association between IL-2 and MLT in patients with advanced tumours of the digestive tract, which are generally less responsive to IL-2 alone (Dillman *et al.*, 1991).

Correspondence: P. Lissoni, Divisione di Radioterapia, Ospedale S. Gerardo, 20052 Monza (Milano), Italy.
Received 3 November 1992; and in revised form 26 January 1993.

### Materials and methods

The study included 35 consecutive patients with advanced tumours of the digestive tract (M/F:22/13; median age 55 years, range 38–70), who were admitted to the Hospital of Monza to receive IL-2 plus MLT as a first or second line therapy for their advanced neoplastic disease. Nineteen patients had been previously treated with chemotherapy, whereas the other patients received the immunotherapy as a first line treatment for their advanced disease. Eligibility crtieria included: histologically proven cancer of the digestive tract, no more than one previous chemotherapy, age less than 75 years, Karnofsky's score greater than 30%. Patients with brain metastases, double tumours or important cardiovascular diseases were not included in the study. Histotype was colorectal carcinoma in 14, gastric carcinoma in eight, adenocarcinoma of pancreas in seven, and hepatocarcinoma in six. Visceral lesions as dominant metastasis sites were present in 31/35 patients, while the other four had a locally advanced disease. The experimental protocol was explained to each patient, and informed consent was obtained.

MLT was supplied by Medea Research (Milan, Italy). Human recombinant IL-2 was supplied by Euro-Cetus (Amsterdam, Holland). MLT was given orally at a dose of 50 mg/day in the evening (8.00 p.m.) because of its greater biological activity in the night (Bartsch & Bartsch, 1981). MLT was given every day, starting 7 days before the first IL-2 injection as an induction phase to enhance host biological response to IL-2 (Lissoni *et al.*, 1991). IL-2 was injected subcutaneously at 3 million IU/day at 8.00 p.m. for 6 days/week for 4 consecutive weeks, corresponding to one cycle of therapy. We decided to administer IL-2 subcutaneously because of its lower toxicity in comparison to the intravenous route of administration (Atzpodien *et al.*, 1991). Moreover, we decided to give IL-2 in the evening because of the spontaneous increase in lymphocyte proliferative capacity in this period of the day (Ritchie *et al.*, 1983). In non-progressed patients, a second cycle was given after a rest period of 3 weeks, after that patients followed a maintenance period consisting of 1 week of therapy every month until progression.

Radiological examinations were repeated after each cycle of therapy, then every 2 months. Liver involvement was investigated by CT scan. Clinical response and toxicity were evaluated according to WHO criteria. Complete response (CR) was a complete resolution of all clinically evaluable disease for at least 1 month; partial response (PR) was defined as at least 50% reduction in the sum of the products of the longest perpendicular diameters of measurable lesions for at least 1 month; stable disease (SD) was defined as no objective tumour regression or increase greater than 25%; progressive disease (PD) was defined as at least 25% increase in measurable lesions or the appearance of new lesions. Patients were considered as evaluable when they received at least one complete cycle of therapy.

Routine laboratory tests, including leukocyte count, and electrocardiogram were made before and repeated weekly during IL-2 administration. Moreover, to analyse macrophage activation, serum levels of neopterin were also measured at 1-week intervals, by using the RIA method and commercially available kits (Henning, Berlin–Germany).

Data were statistically evaluated by the Student's *t*-test, analysis of variance according to Newman Keuls test adjusted for a correction factor, and chi-square test.

### Results

Clinical data and response to therapy are reported in Table I. Two patients achieved a CR, the former affected by gastric cancer with liver metastases and the latter by locally advanced hepatocarcinoma (duration: 19+ and 12+ months, respectively). A PR was obtained in six other patients (gastric cancer: 2; hepatocarcinoma: 2; colon cancer: 1; cancer of pancreas: 1). Therefore, the objective tumour regression rate was 8/35 (23%) patients. The objective regression rate was significantly higher in untreated patients than in those

**Table I** Clinical data and response to therapy in 35 patients with advanced neoplasms of the digestive tract treated with IL-2 plus MLT

| | | | | *Clinical response* | | | | |
|---|---|---|---|---|---|---|---|---|
| *Cases* | *Sex* | *Age* | *Sites of disease* | *Response*[a] | *Sites* | *Response Duration (months)* | *Progression sites* | *Survival (months)* |
| *Colorectal adenocarcinoma* | | | | | | | | |
| [b]1 | F | 42 | Liver | PD | – | – | Liver | 4 |
| [b]2 | M | 38 | Peritoneum | PR | Peritoneum | 3 | Peritoneum | 7 |
| [b]3 | F | 69 | Liver | PD | – | – | Liver | 6 |
| [b]4 | M | 63 | Liver, lung | PD | – | – | Liver | 5 |
| [b]5 | M | 69 | Liver, lung | PD | – | – | Brain | 7 |
| [b]6 | F | 43 | Liver, lung | PD | – | – | Liver | 4 |
| [b]7 | F | 42 | Liver, lung | PD | – | – | Liver | 7 |
| [b]8 | F | 40 | Liver | PD | – | – | Liver | 5 |
| [b]9 | M | 55 | Peritoneum | SD | – | 10 | Peritoneum | 14 |
| [b]10 | M | 50 | Liver | PD | – | – | Liver | 6 |
| [b]11 | F | 65 | Liver | SD | – | 12+ | – | 12+ |
| [b]12 | M | 69 | Liver | PD | – | – | Liver | 7 |
| [b]13 | M | 55 | Lung, bone, nodes | SD | – | 5 | Lung | 12+ |
| [b]14 | M | 58 | Bone | PD | – | – | Bone | 9 |
| *Gastric adenocarcinoma* | | | | | | | | |
| [b]1 | M | 56 | Liver | SD | – | 3 | Liver | 9 |
| 2 | F | 65 | Liver | CR | Liver | 19+ | – | 19+ |
| 3 | M | 65 | Liver | PR | Liver | 4 | Liver | 7 |
| 4 | M | 70 | Liver | SD | – | 9+ | – | 9+ |
| [b]5 | M | 66 | Liver | SD | – | 9+ | – | 9+ |
| [b]6 | M | 58 | Liver, lung, bone | PD | – | – | Liver | 2 |
| 7 | M | 49 | Liver | PR | Liver | 3+ | – | 3+ |
| 8 | M | 62 | Liver | PD | – | – | Liver | 4 |
| *Hepatocarcinoma* | | | | | | | | |
| 1 | M | 54 | Liver | PR | Liver | 21+ | – | 21+ |
| 2 | M | 60 | Liver, lung | PD | – | – | Liver | 4 |
| 3 | F | 44 | Liver | CR | Liver | 12+ | – | 12+ |
| 4 | F | 52 | Liver, bone | SD | – | 6 | Liver | 7+ |
| 5 | M | 56 | Liver, bone | SD | – | 6+ | – | 6+ |
| 6 | F | 54 | Liver | PR | Liver | 4+ | – | 4+ |
| *Pancreas adenocarcinoma* | | | | | | | | |
| 1 | M | 61 | Pancreas, liver | SD | – | 3 | Liver | 4 |
| 2 | F | 52 | Pancreas, liver | SD | – | 6 | Liver | 8 |
| [b]3 | M | 61 | Pancreas, liver | PD | – | – | Liver | 2 |
| 4 | F | 65 | Pancreas, liver | SD | – | 4 | Liver | 6 |
| 5 | M | 53 | Pancreas | PR | Pancreas | 4 | – | 4+ |
| [b]6 | F | 48 | Pancreas, liver | PD | – | – | Liver | 5 |
| 7 | M | 64 | Pancreas, liver | PD | – | – | Pancreas | 5 |

[a]CR = complete response; PR = partial response; s.d. = stable disease; PD = progressive disease. [b]Patients pretreated with chemotherapy.

**Table II** Clinical response to IL-2 plus MLT in relation to tumour histotype in 35 patients with advanced cancer of the digestive tract

| | | Clinical response | | | | |
|---|---|---|---|---|---|---|
| *Histotype* | *n* | *CR* | *PR* | *CR + PR* | *s.d.* | *PD* |
| Overall patients | 35 | 2 (6%) | 6 (17%) | 8 (23%) | 11 (31%) | 16 (46%) |
| Colon cancer | 14 | 0 | 1 (7%) | 1 (7%) | 3 (21%) | 10 (71%) |
| Gastric cancer | 8 | 1 (13%) | 2 (25%) | 3 (37%) | 3 (37%) | 2 (25%) |
| Hepatocarcinoma | 6 | 1 (17%) | 2 (33%) | 3 (50%) | 2 (33%) | 1 (17%) |
| Pancreas cancer | 7 | 0 | 1 (14%) | 1 (14%) | 3 (43%) | 3 (43%) |

previously treated with chemotherapy (7/16 *vs* 1/19; $P<0.01$). Eleven other patients (31%) obtained a s.d. (colon cancer: 3; gastric cancer: 3; hepatocarcinoma: 2; cancer of pancreas: 3). The remaining 16/35 (46%) patients rapidly progressed. The mean survival time was significantly higher in responder patients or in those with s.d. than in patients who progressed under treatment ($8.9 \pm 1.1$ *vs* $5.1 \pm 0.5$ months; $P < 0.05$). Clinical response in relation to tumour histotype is reported in Table II. All tumour regressions were documented by CT scan.

Toxicity was low in all patients, and in particular no cardiovascular, pulmonary, renal or haematological complications occurred. Fever higher than 38°C was observed in only 6/35 (17%) patients, but it was limited to the first day of IL-2 injection, which was made during the admission at the hospital, after that patients followed IL-2 administration as a home therapy. The only other side-effect was anorexia, which occurred in 3/35 (8%) patients. On the contrary, a clear improvement in the performance status was obtained in 9/35 (26%) patients.

Lymphocyte and eosinophil mean increase, as evaluated on the first immunotherapeutic cycle, was significantly higher in patients who responded or had a s.d. than in those progressing under treatment as shown in Table III. On the contrary, mean increase in serum levels of neopterin was significantly higher in progressed patients than in those with response or s.d. ($6.8 \pm 0.7$ *vs* $2.6 \pm 0.3$ ng ml$^{-1}$; mean ± s.e., $P < 0.01$).

**Table III** Increase (mean ± s.e.) in lymphocyte and eosinophil number (n mm$^{-3}$) in relation to the clinical response to IL-2 plus MLT in 35 patients with advanced tumours of the digestive tract

| *Patients* | *n* | *Lymphocytes* | *Eosinophils* |
|---|---|---|---|
| Patients with response or stable disease | 19 | $1690 \pm 210^{a}$ | $1320 \pm 110^{a}$ |
| Progressed patients | 16 | $690 \pm 80$ | $540 \pm 50$ |

$^{a}P < 0.01$ *vs* progressed patients.

### Discussion

This experimental clinical study shows that IL-2 at very low doses is able to determine tumour progressions in advanced neoplasms of the digestive tract when it is associated with the pineal hormone MLT. Gastric cancer and hepatocarcinoma would seem the gastrointestinal neoplasms more responsive to the immunotherapy with IL-2 plus MT. Since IL-2 alone is generally less effective in the treatment of gastrointestinal tumours (Dillman *et al.*, 1991), these results would suggest that MLT may potentiate IL-2 antitumour activity in humans, as previously observed in lung cancer (Lissoni *et al.*, 1992). The mechanisms by which MLT could synergise with IL-2 have still to be better defined; however, they include at least in part the modulation of suppresive events occurring during the immunotherapy (Lissoni *et al.*, 1991). In any case, randomised clinical trials with IL-2 *vs* IL-2 plus MLT will be required to define the real role of MLT as a possible agent to extent the spectrum of IL-2 anti-tumour efficacy in humans.

As far as the relation between biological effects and clinical response is concerned, this study would suggest that the inhibitory control of tumour growth, obtained with IL-2 plus MLT, is mediated by lymphocytes and eosinophils, whereas the activation of macrophages, as documented by neopterin increase, would negatively influence the efficacy of the immunotherapy. This consideration is supported by the evidence of a greater increase in lymphocyte and eosinophil number and of a lower rise in neopterin levels in patients with response or s.d. than in the progressed ones. The importance of host biological response in mediating the efficacy of the immunotherapy is also suggested by the lower response rate in patients previously treated with chemotherapy than in the untreated ones. This finding could depend on chemotherapy-induced damage of bone marrow, which is the main source of cytotoxic anticancer cells. Therefore, an eventual immunotherapeutic treatment with IL-2 and MLT would have to precede the chemotherapy in future possible chemoimmunotherapeutic combinations of advanced neoplasms of the digestive tract.

In conclusion, this study shows that the immunotherapy with low-dose IL-2 and the pineal hormone MLT may represent a new effective and well tolerated therapy of advanced tumours of the digestive tract, apparently mainly in gastric cancer and hepatocarcinoma.

### References

ATZPODIEN, J., KÖRFER, A., EVERS, P., FRANKS, C.R., KNÜVERHOPF, J., LOPEZ-HÄNNINEN, E., FISCHER, M., MOHR, H., DALLMANN, I., HADAM, M., POLIWODA, H. & KIRCHNER, H. (1990). Low-dose subcutaneous recombinant interleukin-2 in advanced human malignancy: a phase II outpatient study. *Mol. Biother.*, **2**, 18-26.

BARTSCH, H. & BARTSCH, C. (1981). Effect of melatonin on experimental tumors under different photoperiods and times of administration. *J. Neural Transm.*, **52**, 269-275.

CUNNINGHAM, D., MANSI, J., FORD, H.T., NASH, A.T. & MENZIES-GOW, N. (1991). Epirubicin, cisplatin and 5-fluorouracil (ECF) is highly effective in advanced gastric cancer. *Proc. ASCO*, **10**, 412.

DILLMAN, R.O., OLDHAM, R.K., TAUER, K.W., ORR, D.W., BARTH, N.M., BLUMENSCHEIN, G., ARNOLD, J., BIRCH, R. & WEST, W.H. (1991). Continuous interleukin-2 and lymphokine-activated killer cells for advanced cancer: a national biotherapy study group trial. *J. Clin. Oncol.*, **9**, 1233-1240.

GRIMM, E.A., MAZUMDER, A., ZHANG, H.Z. & ROSENBERG, S.A. (1982). Lymphokine-activated killer cell phenomenon. *J. Exp. Med.*, **155**, 1823-1841.

LISSONI, P., BARNI, S., CRISPINO, S., TANCINI, G. & FRASCHINI, F. (1989). Endocrine and immune effects of melatonin therapy in metastatic cancer patients. *Eur. J. Cancer Clin. Oncol.*, **25**, 789-795.

LISSONI, P., TISI, E., BARNI, S., ARDIZZOIA, A., ROVELLI, F., RESCALDANI, R., BALLABIO, D., BENENTI, C., ANGELI, A., TANCINI, G., CONTI. A. & MAESTRONI, G.J.M. (1992). Biological and clinical results of a neuroimmunotherapy with interleukin-2 and the pineal hormone melatonin a first line treatment in advanced non-small cell lung cancer. *Br. J. Cancer*, **66**, 155–158.

LISSONI, P., TISI, E., BRIVIO, F., ARDIZZOIA, A., CRISPINO, S., BARNI, S., TANCINI, G., CONTI, A. & MAESTRONI, G.J.M. (1991). Modulation of interleukin-2-induced macrophage activation in cancer patients by the pineal hormone melatonin. *J. Biol. Regul. Homeost. Agents*, **5**, 154–156.

MAESTRONI, G.J.M., CONTI, A. & PIERPAOLI, W. (1986). Role of the pineal gland in immunity. Circadian synthesis and release of melatonin modulates the antibody response and antagonizes the immunosuppressive effect of corticosterone. *J. Neuroimmunol.*, **13**, 19–25.

MCCANN, S.M., ONO, N., KHORRAM, O., KENTROTI, S. & AGUILA, C. (1987). The role of brain peptides in neuroimmunomodulation. *Ann. N.Y. Acad. Sci.*, **496**, 178–185.

MATHEWS, P.M., FROELICH, C.J., SIBBIT, W.L. & BANKHURST, A.A. (1983). Enhancement of natural cytotoxicity by β-endorphin. *J. Immunol.*, **130**, 1658–1662.

RITCHIE, A.W., OSWALD, I., MICKLEM, H.S., BOYD, J.E., ELTON, R.A., JAZWINSKA, E. & JAMES, K. (1983). Circadian variation of lymphocyte subpopulations: a study with monoclonal antibodies. *Br. J. Med.*, **286**, 1773–1776.

ROSENBERG, S.A., LOTZE, M.T., MUUL, L.M., CHANG, A.E., AVIS, F.P., LEITMAN, S., LINEHAN, W.M., ROBERTSON, C.N., LEE, R.E., RUBIN, J.T., SEIPP, C.A., SIMPSON, C.G. & WHITE, D.E. (1987). A progress report on the treatment of 157 patients with advanced cancer using lymphokine-activated killer cells and interleukin-2 or high-dose interleukin-2 alone. *N. Engl. J. Med.*, **316**, 889–897.